# Autoantibodies Against Perilipin 1 as a Cause of Acquired Generalized Lipodystrophy

**DOI:** 10.3389/fimmu.2018.02142

**Published:** 2018-09-19

**Authors:** Fernando Corvillo, Verónica Aparicio, Alberto López-Lera, Sofía Garrido, David Araújo-Vilar, María P. de Miguel, Margarita López-Trascasa

**Affiliations:** ^1^La Paz University Hospital Research Institute, Madrid, Spain; ^2^Center for Biomedical Network Research on Rare Diseases, Madrid, Spain; ^3^Cell Engineering Laboratory, La Paz University Hospital Research Institute, Madrid, Spain; ^4^Thyroid and Metabolic Diseases Unit (U.E.T.eM.), Department of Medicine, Universidad de Santiago de Compostela, Santiago de Compostela, Spain; ^5^Departamento de Medicina, Universidad Autónoma de Madrid, Madrid, Spain

**Keywords:** generalized lipodystrophy, perilipin, autoimmunity, lipolysis, Lawrence syndrome

## Abstract

Acquired generalized lipodystrophy (AGL) is a rare condition characterized by an altered distribution of adipose tissue and predisposition to develop hepatic steatosis and fibrosis, diabetes, and hypertriglyceridemia. Diagnosis of AGL is based on the observation of generalized fat loss, autoimmunity and lack of family history of lipodystrophy. The pathogenic mechanism of fat destruction remains unknown but evidences suggest an autoimmune origin. Anti-adipocyte antibodies have been previously reported in patients with AGL, although their involvement in the pathogenesis has been poorly studied and the autoantibody target/s remain/s to be identified. Using a combination of immunochemical and cellular studies, we investigated the presence of anti-adipocyte autoantibodies in patients with AGL, acquired partial lipodystrophy, localized lipoatrophy due to intradermic insulin injections or systemic lupus erythematosus. Moreover, the impact of anti-adipocyte autoantibodies from AGL patients was assessed in cultured mouse preadipocytes. Following this approach, we identified anti-perilipin 1 IgG autoantibodies in the serum of patients with autoimmune variety-AGL, but in no other lipodystrophies tested. These autoantibodies altered the ability of perilipin 1 to regulate lipolysis in cultured preadipocytes causing abnormal, significantly elevated basal lipolysis. Our data provide strong support for the conclusion that perilipin 1 autoantibodies are a cause of generalized lipodystrophy in these patients.

## Introduction

Acquired generalized lipodystrophy (AGL), or Lawrence syndrome (ORPHA:79086), is a rare disease that usually develops during childhood and adolescence. It is characterized by a selective loss of adipose tissue from large areas of the body, particularly the face, arms, and legs (1). Reported in more than 100 cases with a male-to-female ratio of 1:3, most patients with AGL have fasting and/or postprandial hyperinsulinemia, severe hepatic steatosis, diabetes mellitus, hypertriglyceridemia, and low serum levels of high-density lipoprotein cholesterol, leptin, and adiponectin (1, 2). Approximately 25% of patients debut with an episode of panniculitis. Another 25% of cases present associated autoimmune diseases, particularly juvenile dermatomyositis. In the remaining patients, the underlying mechanism of fat loss is not clear (idiopathic variety). The metabolic complications are typically less severe in patients with the panniculitis-associated phenotype compared with the other two subtypes (3).

The pathogenesis of AGL can vary, and the precise mechanisms accounting for fat loss remain unknown. In patients with the panniculitis and autoimmune varieties, loss of adipose tissue is presumed to be due to either a cell-mediated or an antibody-mediated autoimmune process or both (1). The only evidence of anti-adipocyte autoantibodies was reported in a 33-year-old male patient with AGL, insulin-resistant diabetes, and acanthosis nigricans (4). The authors demonstrated IgG deposits around the adipocytes using direct immunofluorescence on the patient's subcutaneous biopsy. However, the target autoantigen/s have not been yet identified. In addition, some patients have been reported to have chronic hepatitis with autoimmune features and low complement C4 levels, suggesting involvement of the complement classical pathway in the pathogenesis of fat loss (5, 6). Unlike in AGL, scientific evidences exist for the involvement of complement-dependent cytotoxicity in the lysis of adipocytes in patients with acquired partial lipodystrophy (APL) (7). A recent work presented three males diagnosed of generalized lipodystrophy, with subsequent development of pilocytic astrocytoma (8). All of them gained body fat or weight after surgical removal of the tumor and/or chemotherapy. The authors hypothesized that the tumor secreted anti-adipocyte antibodies, which could have induced adipocyte lysis.

Here, we demonstrate the presence of anti-adipocyte antibodies predominantly directed against perilipin 1 (PLIN1) in patients with AGL. PLIN1 is the most abundant adipocyte-specific protein that coats lipid droplets, which is required for optimal lipid incorporation and release from the droplet. Mutations in the gene encoding PLIN1 have been described in a group of patients with familial partial lipodystrophy type 4 (FPLD4) (9, 10). Moreover, we show that anti-PLIN1 autoantibodies, but not IgG from healthy donors, significantly increased basal lipolysis in cultured preadipocytes. These findings reveal a novel autoimmune mechanism accounting for the loss of adipose tissue in patients with AGL.

## Materials and methods

### Patients

Five patients (4 F/1 M) were diagnosed with AGL on the basis of fat loss during childhood or adulthood affecting large areas of the body, and having ruled out other causes of weight loss. Congenital generalized lipodystrophy was also excluded based on the natural course of the disease, clinical features, age at onset, and discarding pathogenic variants in both the CGL-related genes AGPAT2, BSCL2, CAV1, and PTRF, and also FPL-related genes LMNA and PPARG. No consanguinity was reported in any case. We used the criteria for designating AGL variety described by Misra and Garg (3): I) Autoimmune variety: AGL follows an autoimmune diseases or the presence of autoantibodies; II) Panniculitis variety: Tender subcutaneous nodules that herald the onset of AGL; III) Idiopathic variety: No history of autoimmune disease or panniculitis. Physical, anthropometric, and clinical features from patients with AGL are shown in Table 1.

**Table 1:** Clinical features of patients with AGL.

**Variable**	**AGL1**	**AGL2**	**AGL3**	**AGL4**	**AGL5**	**Reference range**
Sex	Female	Male	Female	Female	Female	
Age (years)	33	10	13	51	33	
Lipodystrophy onset (years)	5	2	3	5	31	
AGL subtype	Autoimmune	Autoimmune	Autoimmune	Idiopathic	Idiopathic	
BMI (kg/m^2^)	17.3	15^*^	16^¥^	19.2	15.2	18–25
% total fat^£^	8	NA	11	20.5	10.2	27.2–33.3
Acanthosis	Yes	No	Yes	No	No	
Glucose (mg/dL)	121	104	98	92	81	60–110
Insulin (μU/mL)	Insulin-treated	26	275.9	114	3.7	2.6–24.9
Diabetes	Yes	No	No	No	No	
HbA_1c_ (%)	6.8	5.3	5.8	5.4	5.4	4.0–5.5
Hypoleptinemia	Yes	Yes	Yes	Yes	Yes	
Triglycerides (mg/dL)	79	104	718	138	69	25–115
Fatty liver^†^	No	No	Yes	No	No	
ALT (UI/L)	50.6	55.8	131	27	26	5–31
AST (UI/L)	34.6	35.4	77	18	20	5–32
Autoimmunity	T1DM	Anti-GAD Anti-TPO	APCA	No	No	
Immunosuppressant treatment	No	No	No	No	No	
IgG (mg/dL)	1,280	1,120	1,415	988	1,270	725–1,900
IgA (mg/dL)	135	139	196	122	230	50–340
IgM (mg/dL)	97.2	92.2	193	94	86.4	45–280
C3 (mg/dL)	112	109	170	143	97.1	75–135
C4 (mg/dL)	17.1	24.2	35	33.9	16.8	14–60

BMI, body mass index; ALT, Alanine aminotransferase; AST, Aspartate aminotransferase; T1DM, type 1 diabetes mellitus; Anti-GAD, anti-glutamic acid decarboxylase antibody; APCA, anti-parietal cell antibodies; Anti-TPO, anti-Thyroperoxidase antibody; NA, not available.

* BMI among 13.7-18.5 is considered normal for 10 years old children.

¥ BMI among 14.9-20.8 is considered normal for 13 years old children.

£ Percentage of total fat was measured using dual-energy x-ray absorptiometry.

† *Liver steatosis was assessed by means of ultrasonography*.

### Biological samples

We collected serum and plasma samples from five patients with AGL. As disease control, we used serum from 8 patients diagnosed with acquired partial lipodystrophy (APL), according to the criteria by Araujo-Vilar and Santini (11). Additionally, we tested serum from 11 patients with localized lipoatrophy due to intradermic insulin injections (I-LA), 10 patients with systemic lupus erythematous (SLE), and 20 healthy volunteers. Serum and EDTA plasma samples were obtained under standard conditions upon informed consent from the donors; blood was collected in plain tubes, allowed to clot at room temperature, and centrifuged for 10 min at 4°C. Serum and plasma were then aliquoted and stored frozen at −80°C until their use.

Adipose tissue from four female donor patients (ages 22 to 53, mean 40 years of age, and BMI range 42.60 to 19.57, mean 31.3) undergoing elective liposuction was obtained for isolation of hMSCs. Patients were otherwise healthy and received no drug therapy. Active infection by HIV, hepatitis C virus, and syphilis was ruled out by serological analyses. For histological and western blot analyses, the subcutaneous adipose tissue was biopsied. The tissues were frozen and included in optimal cutting temperature compound for histological studies. Adipose tissue was lysed to extract total protein and kept frozen at −80°C until used.

All procedures involving human participants were performed in accordance with the standards of the Ethics Review Panel of La Paz University Hospital (Madrid, Spain) and with the 2013 Helsinki declaration and its later amendments or comparable ethical standards. All participants provided written informed consent for participation in the study and for the publication of their clinical and biochemical information.

### Protein extraction from adipose tissue

Total protein extraction from subcutaneous white adipose tissue was performed using Protein extraction kit (101bio) following manufacturer's instructions. The extracts were depleted of albumin and IgG with ProteoPrep® Immunoaffinity Albumin & IgG Depletion Kit from Sigma. Subsequently, protein concentration was determined using Bradford assay and the samples were stored at −80°C until used.

### Western blotting

Ten micrograms of adipose tissue extract were run on precast 4–20% Tris-HCl Ready Gels (Bio-Rad Laboratories) in the presence of Tris-glycine-SDS running buffer. Proteins were transferred to a polyvinylidene fluoride membrane using the iBlot® 7-Min Blotting System (Thermo Fisher), following the manufacturers indications. Membranes were blocked for 1 h at room temperature in assay buffer containing 10 mM Tris-HCl, pH 7.6, 5% of fetal calf serum, 150 mM NaCl and 1% of Tween-20.

All incubations with human serum were performed for overnight at 4°C at a 1:100 dilution in assay buffer. The membranes were washed three times with 0.1% of Tween-20-containing TBS buffer. Negative samples for anti-adipocyte antibodies were also tested at 1:100 and 1:50 dilutions but remained negative. The washed membranes were incubated with alkaline phosphatase-conjugated anti-human IgG (1:8,000; 1 h at room temperature) from Jackson Immunoresearch diluted in assay buffer. To detect PLIN1 in adipose tissue extracts, the membranes were incubated with an Antigen Affinity-purified sheep polyclonal anti-PLIN1 antibody directed to the peptide spanning Thr8-Ala145 (catalog. #AF6615, from R&D) at 1 μg/mL. Additionally, a rabbit polyclonal anti-PLIN1 antibody targeting amino acids surrounding Asp418 (named D418 for us, catalog. #3470, from Cell Signaling Technology) was used to compare autoantibody reactivity against the C-terminal domain of PLIN1. In both experiments appropriate alkaline phosphatase-conjugated antibodies (Sigma) were used and the blots were developed with 5-bromo-4-chloro-3-indolyl-phosphate/nitro blue tetrazolium (BCIP/NBT, from VWR).

To demonstrate the specificity of the autoantibodies in patients with AGL, serum samples were preabsorbed at 1:100 dilutions with 14 μg of recombinant PLIN1 (OriGene, transcript variant 1, RefSeq: NP_002657) overnight at 4°C. As control sample, an identical volume of PBS was mixed with the patient's serum. After the blockage step, tissue extracts were blotted with either treated or non-treated patient's serum as described above.

### Immunohistological studies

Cryosections of normal human subcutaneous adipose tissue obtained from healthy donors were fixed for 10 min in acetone and blocked with 10% bovine serum albumin. We used serum as the primary antibody at a 1:10 dilution, followed by a fluorescein isothiocyanate-conjugated rabbit anti-human IgG (1.5 μg/mL, F0315, from Dako) to detect immunoglobulin deposition. Immunofluorescence images were obtained with an Olympus camera (Olympus Corporation) mounted on an Olympus microscope and analyzed with DP software (Olympus).

### Human mesenchymal stem cells isolation

Isolation of hMSC was performed as described by Fuentes-Julián et al. (12). Briefly, liposuction-derived adipose tissue was washed with PBS and digested with 0.09% collagenase I in PBS (Gibco) for 45 min at 37°C under gentle agitation. The reaction was stopped with FBS (Gibco) and centrifuged at 300 g for 10 min to separate the stroma vascular fraction of adipose tissue of the pelleted cells from the floating adipocytes. Pellets were treated with erythrocyte lysis buffer (160 mM NH_4_Cl; 10 mM KHCO_3_; 1 mM EDTA; all from Sigma) for 15 min at room temperature, the erythrocytes-free cell fraction was washed with PBS and seeded at a 1 × 10^6^ cells per plate density on 10-cm plates (Costar®, Corning) in standard DMEM (Gibco), supplemented with 10% FBS and 1% penicillin-streptomycin (Sigma).

### *In vitro* adipogenesis

Human MSCs obtained from adipose tissue were differentiated into preadipocytes in an adipogenic culture medium containing: DMEM supplemented with 10% FBS, 1% penicillin-streptomycin, 500 μM isobutylmethylxanthine (IBMX), 1 μM dexamethasone, 1 μM indomethacin and 10 μg/mL of insulin (Lilly). The medium was refreshed every 48 h. Three weeks after differentiation, adipogenesis was completed as judged by the enrichment in big-sized lipid droplets.

3T3-L1 cell line was differentiated into preadipocytes for 2 weeks. Cells were cultured in DMEM medium supplemented with 10% FBS, 1% penicillin-streptomycin, 500 μM IBMX, 1 μM dexamethasone and 10 μg/mL of insulin. After 48 h of culture, medium was replaced for DMEM medium supplemented with 10% FBS, 1% penicillin-streptomycin and 10 μg/mL of insulin for another 48 h. Differentiation was subsequently completed by incubation in 10% FBS, 1% penicillin-streptomycin-supplemented DMEM for 10 days.

### Immunocytochemistry

Fixed (4% paraformaldehyde) cultured preadipocytes were washed with PBS, blocked and permeabilized with 0.3% Triton X-100 (Sigma) and 1% BSA-supplemented PBS. Cells were overnight incubated at 4°C with serum at 1:200 dilutions in a 0.3% Triton X-100 and 0.5% BSA-containing PBS buffer. After three washing steps with PBS, the cells were incubated with a FITC-conjugated polyclonal rabbit F(ab′)_2_ anti-human IgG (Dako) (1:250) for 1 h at room temperature. To detect PLIN1, cell cultures were incubated overnight at 4°C with a rabbit anti-human PLIN1 antibody (catalog number #3470, from Cell Signaling Technology) at 1:100, and a convenient biotin-conjugated secondary antibody (1:1000, Vector Laboratories). The reaction was developed using avidin-Texas Red conjugated (1:1000, A-2006, from Vector Laboratories). Plates were then mounted with DAPI-containing Vectashield (Vector Laboratories). To evaluate if AGL patients' sera recognize murine PLIN1, 3T3-L1-derived preadipocytes were processed as described above for cultured human preadipocytes.

### Strategy to identify the antigen

To determine the identity of the antigen targeted by the autoantibodies in patients with AGL, a comprehensive review of the literature (13, 14) and open-access databases (Courtesy of Human Protein Atlas, www.proteinatlas.org) (15, 16) was performed. Selected lipid droplet-associated proteins are summarized in Table 2. The selection criteria used to prioritize the antigen candidates were (a) similar molecular weight; (b) major expression in white adipose tissue and (c) previous associations with other lipodystrophy diseases.

**Table 2:** Candidate antigens in acquired generalized lipodystrophy.

**Gene**	**Gene description**	**RNA expression (TPM)**	**Molecular weight (kDa)**	**Subcellular location**	**Pathology (MIM number)**
*FABP4*	Fatty acid binding protein 4	3642.8	14.7	Cytoplasm/Nucleus	–
*PLIN1*	Perilipin 1	1011.2	56.0	Lipid droplet	Familial partial lipodystrophy type 4 (# 613877)
*ADIPOQ*	Adiponectin	935.8	26.4	Secreted	Adiponectin deficiency (#612556)
*LIPE*	Lipase E, hormone sensitive type	868.9	116.6	Cytoplasm	Familial partial lipodystrophy type 6 (# 615980)
*PLIN4*	Perilipin 4	810.9	136.0	Lipid droplet	–
*CIDEC*	Cell death-inducing DFA-like effector c	760.1	26.8	Lipid droplet	Familial partial lipodystrophy type 5 (#615238)
*PNPLA2*	Patatin-like phospholipase domain-containing protein 2	419.8	55.3	Lipid droplet	Neutral lipid storage disease with myopathy (#610717)
*CFD*	Factor D (adipsin)	378.7	27.0	Secreted	Factor D deficiency (#613912)
*LEP*	Leptin	321.7	18.6	Secreted	Morbid obesity due to leptin deficiency (#614962)
*CIDEA*	Cell death-inducing DFA-like effector a	220.9	24.7	Lipid droplet	–
*ACACB*	Acetyl-CoA carboxylase beta	194.0	276.5	Mitochondria	–
*GYG2*	Glycogenin 2	161.7	55.2	Cytoplasm/Nucleus	
*TUSC5*	Tumor suppressor candidate 5	109,9	19.3	Plasma membrane	–
*LGALS12*	Lectin, galactoside-binding, soluble 12	89.7	37.5	Mitochondria/Nucleus	–
*ACVR1C*	Activin A receptor type 1C	33.9	54.9	Plasma membrane	–

*TPM, transcripts per million; MIM, mendelian inheritance in man*.

### Anti-PLIN1 autoantibody detection by enzyme-linked immunosorbent assay (ELISA)

ELISA microtiter plates (Medisorb, Nunc, VWR) were coated with 100 ng/well of recombinant PLIN1 (OriGene) in carbonate-bicarbonate buffer pH 9.6 (overnight, 4°C). Plates were blocked with PBS supplemented with 0.05% Tween-20 and 3% non-fat milk and washed with PBS-Tween 0.1%. All samples were added at 1:100 dilutions in assay buffer (1 h at 37°C) and run in duplicates. Upon washing, anti-PLIN1 autoantibodies were detected using a peroxidase-conjugated anti-human IgG at 1:10,000 (Jackson Immunoresearch). Color was developed using (2,2′-Azinobis [3-ethylbenzothiazoline-6-sulfonic acid]-diammonium salt (ABTS, from VWR) and absorbance was measured at 405 nm.

### Measurement of lipolytic activity

Mouse 3T3-L1 preadipocytes were cultured in 10% FBS, 1% penicillin-streptomycin-containing DMEM supplemented with 0.0005 μCi/μl of palmitic acid-[9,10-3H (N)] (PerkinElmer), overnight at 37°C and 5% CO_2._ The next day, cells were washed three times with PBS to remove the excess of isotope.

Plasma IgG was purified by affinity chromatography in protein G columns (ABT). In order to assess the effect of anti-PLIN1 autoantibodies on the lipolytic pathway, IgG purified from either AGL patients or healthy donors was used. For the measurement of basal lipolysis, preadipocytes were cultured in MEM medium (Fisher Scientific) containing 1% penicillin-streptomycin, 2% fatty acid-free BSA (Sigma). A 10 μM isoprenaline solution (Sigma) was added to this medium for the valoration of stimulated lipolysis. Additionally, 300 μg of IgG purified from either patients with autoantibodies or healthy donors were added to the medium. Conditioned medium was collected at 60-, 120-, and 180-min time points and analyzed for radiolabeled palmitic acid release in a liquid scintillation analyzer (Model Tri-Carb® 2800TR; PerkinElmer) after addition of Opti-Fluor O (PerkinElmer). Additionally, other experiments were carried out at 180 as the final time point, using 10 μg of human PLIN1 Antigen Affinity-purified Polyclonal Antibody (#AF6615, from R&D) as a positive control.

### Statistical analyses

Statistical calculations were performed with Prism version 6.01 (GraphPad Software). Bonferroni's multiple comparison test was used for comparisons of all the groups. A *P* < 0.05 was considered statistically significant in all analyses.

## Results

### Serum from patients with AGL shows reactivity against adipocyte lipid droplets

**Figure 1:**
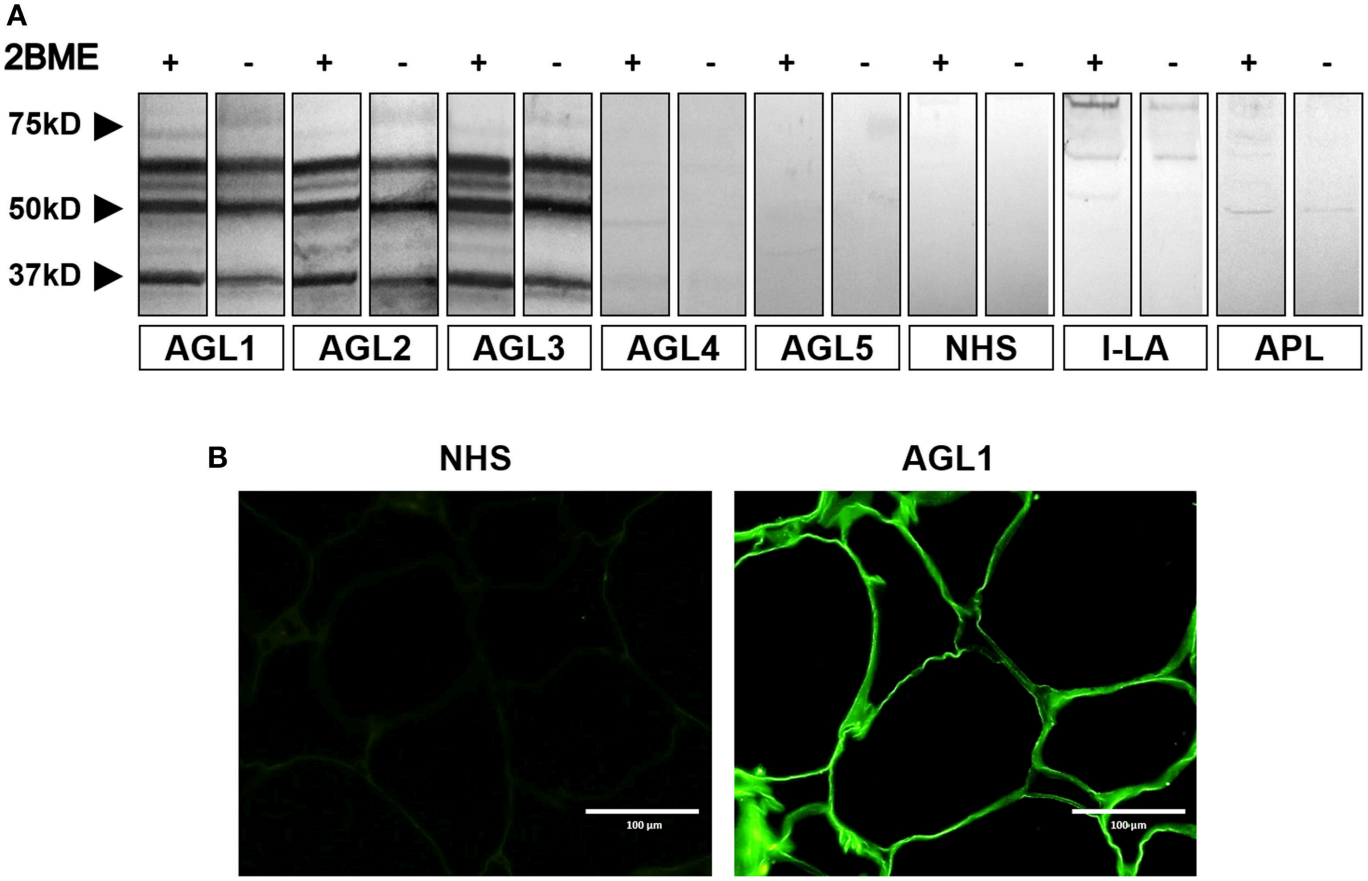
Screening for autoantibodies against adipose tissue in serum samples obtained from patients with acquired lipodystrophies. **(A)** Results of a western blot screening performed on human adipose tissue extracts, under reducing (2BME +) or non-reducing (2BME –) conditions, incubated with serum samples from five patients with acquired generalized lipodystrophy (AGL1 through AGL5), one patient with localized lipoatrophy due to intradermic insulin injections (I-LA), one patient with acquired partial lipodystrophy (APL) and one healthy donor (NHS). **(B)** Indirect immunofluorescence of cryosections of human white adipose tissue with serum from one NHS (left) and serum from one patient with AGL (right), incubated at 1:10 dilutions. IgG deposition on adipocytes (green) was detected with AGL sera as a source of autoreactive immunoglobulins. Scale bars correspond to 100 μm.

Normal adipose tissue extracts were evaluated by western blot by using AGL sera for the detection of transferred proteins. A complex band pattern was observed (three bands at 60, 55, and 37 kDa) in samples from three patients with the autoimmune variety of the disease (designated AGL1, AGL2, and AGL3, Table 1) under reducing and nonreducing conditions (Figure 1A). In contrast, no reactivity was observed in sera from 20 healthy donors, 8 patients with APL and 11 patients with I-LA. The two negative AGL cases in the series (AGL4 and AGL5) were retested at 1:50 serum dilutions, but remained negative. Furthermore, western blot assays were also developed using adipose tissue extracts from different healthy donors obtaining identical results (Figure S1).

Additionally, we used frozen sections of normal adipose tissue to assess serum IgG binding to adipocytes. The results of indirect immunofluorescence confirmed that IgG from those patients with AGL presenting the autoimmune variety of the disease (AGL1, AGL2, and AGL3), but not that from AGL patients with the idiopathic variety (AGL4 and AGL5), reacted against adipocytes (Figure 1B and Table 1). Considering that mature white adipocytes store triglycerides within a large, single lipid droplet, which occupies up to 90% of the cell volume, the precise site of IgG reactivity (i.e., plasmatic membrane or lipid droplet membrane) could not be determined in this experimental setting.

**Figure 2:**
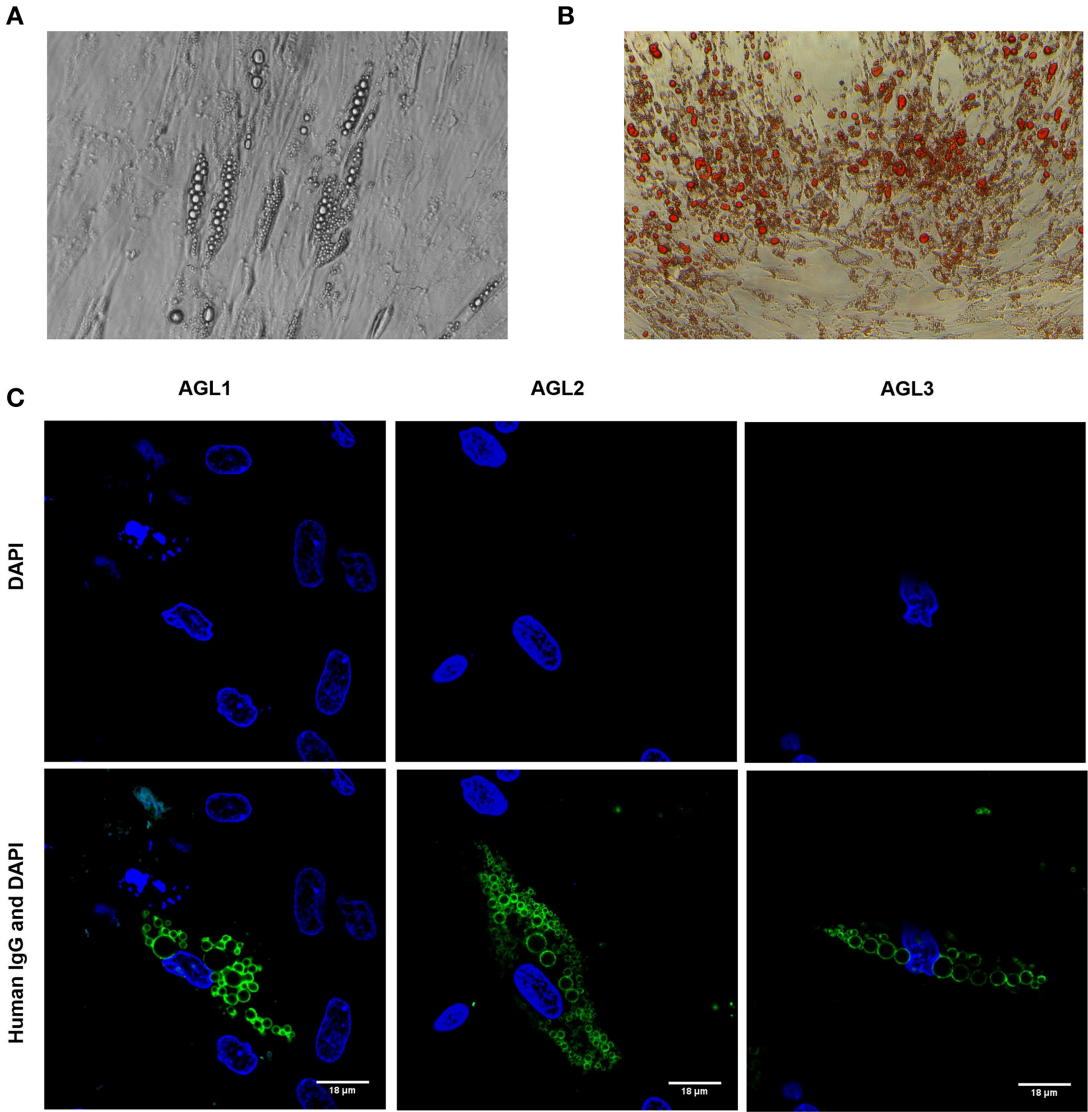
Autoantibody binding to cultured preadipocytes **(A)** Images of phase contrast microscopic picture of cultured hMSCs from healthy donors subjected to adipocyte differentiation for 3 weeks. **(B)** Oil Red O-stained cell culture dishes were photographied using a digital camera. Preadipocyte monolayers images were obtained with an inverted-phase contrast microscope at a magnification of 200x. **(C)** Results of confocal microscopic analysis of human preadipocyte cell cultures. Serum IgG from patients with AGL reacts with an antigen localized at the lipid droplet membrane. Scale bars correspond to 18 μm. DNA was stained with 4′,6-diamidino-2-phenylindole (DAPI, blue). IgG binding was detected using FITC-conjugated rabbit anti-human IgG (green).

In order to establish the subcellular location and specific antigen/s bound by autoreactive IgG in these patients, adipose tissue-derived human mesenchymal stem cells (hMSCs) were differentiated into preadipocytes (Figures 2A,B). After 3 weeks of adipogenic induction, most cells presented intracellular lipid vacuoles strongly bound by IgG from patients with AGL (Figure 2C), thus demonstrating that the target autoantigen is a lipid droplet component.

### PLIN1 is targeted by autoantibodies in patients with AGL

The search for hypothetical autoantigen candidates was performed *in silico* as described in the Methods section and the main candidates are shown in Table 2. Based on its significant expression on the lipid droplet, compatible molecular weight and pathological significance, the first interrogated candidate was PLIN1. PLIN1 is one of the most abundant proteins on the lipid droplet surface of mature white adipocytes (17), has a molecular weight of 55.9 kDa, and heterozygous PLIN1 mutations are associated with FPLD4 (9, 10).

**Figure 3:**
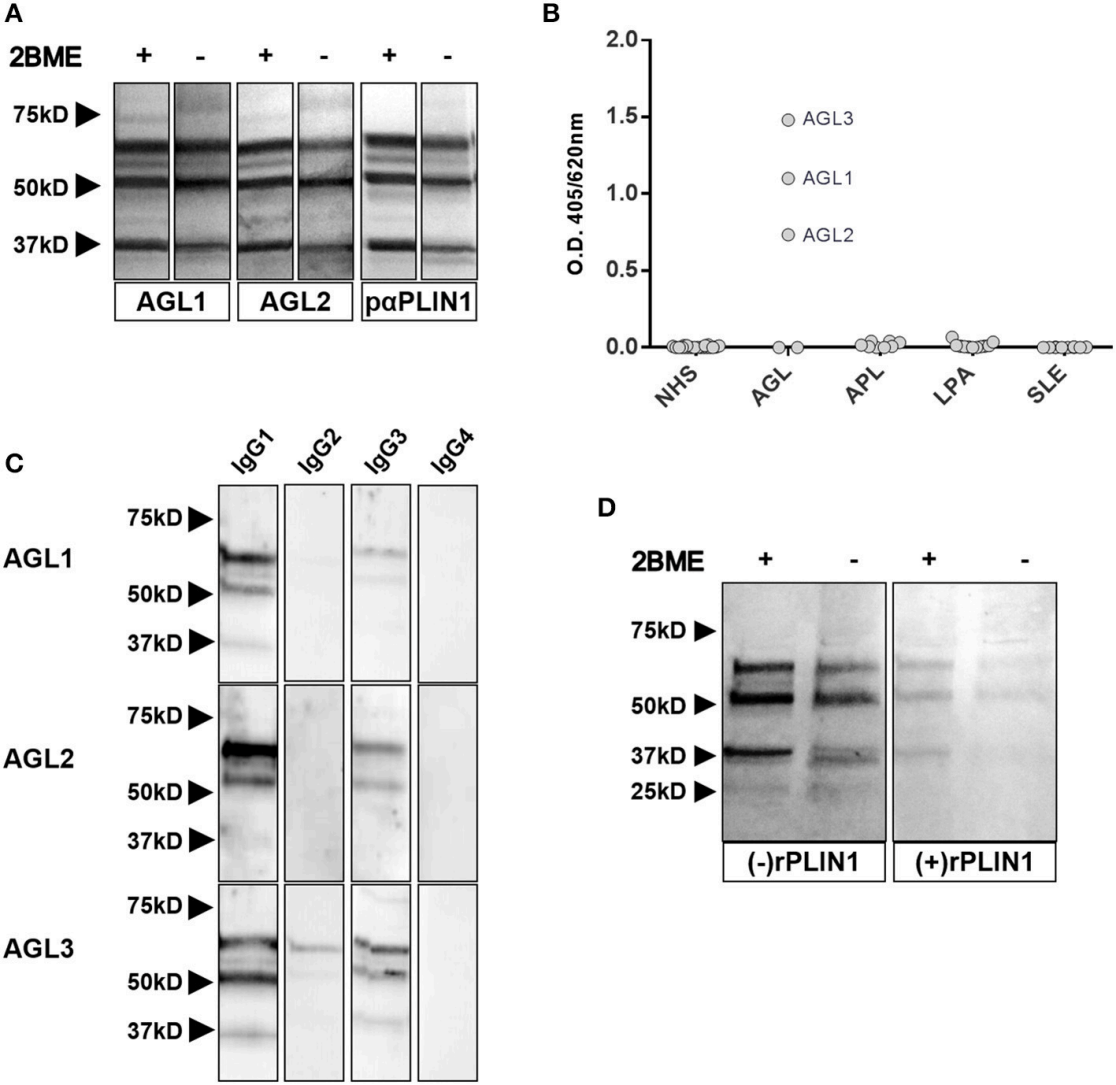
Characterization of anti-PLIN1 autoantibodies in patients with AGL. **(A)** Equal amounts of human adipose tissue were electrophoresed under reducing (2BME +) and nonreducing (2BME –) conditions. Membrane was incubated with either reactive serum samples from two patients with AGL as source of antibodies or a polyclonal anti-PLIN1 IgG (#AF6615) and detected with appropriate secondary antibodies. **(B)** Results of ELISA analyses demonstrating that IgG from three patients with AGL (ALG1, AGL2, and ALG3) binds to recombinant human PLIN1. Neither serum from healthy donors (*n* = 20, NHS), nor from patients with acquired partial lipodystrophy (*n* = 8, APL), localized lipoatrophy due to intradermic insulin injections (*n* = 11, I-LA), and systemic lupus erythematosus (*n* = 10, SLE) reacted against recombinant human PLIN1 on ELISA assay. Samples were run in duplicates. Each point represents the mean value of optical density (O.D.) for each sample. **(C)** White adipose tissue extracts were blotted with serum samples from three anti-PLIN1-positive patients (AGL1 through AGL3), followed by detection with antibodies specific for each human IgG subclass (1 through 4), and peroxidase conjugated anti-subclass-specific IgG antibodies**. (D)** Blotted human adipose tissue proteins were revealed using serum from a patient with anti-PLIN1 autoantibodies either pre-incubated (right) or not (left) with recombinant human PLIN1. PLIN1 detection is blocked by the recombinant protein, thus demonstrating specificity.

Western blot assays with commercial polyclonal anti-PLIN1 antibody (#AF6615) performed on adipose tissue lysates yielded a band pattern identical to that obtained with serum from patients with AGL (Figure 3A). In addition, we performed an ELISA assay to detect anti-PLIN1 specific antibodies in the serum of patients with AGL, I-LA, APL, SLE and healthy donors, using recombinant human PLIN1 as a target antigen. Only AGL1, AGL2, and AGL3 (all of them with previous positive results in western blotting) had detectable anti-PLIN1 IgG autoantibodies (Figure 3B). Anti-PLIN1 autoantibodies were found to be predominantly IgG1, although smaller amounts of other subclasses were also detected (Figure 3C).

**Figure 4:**
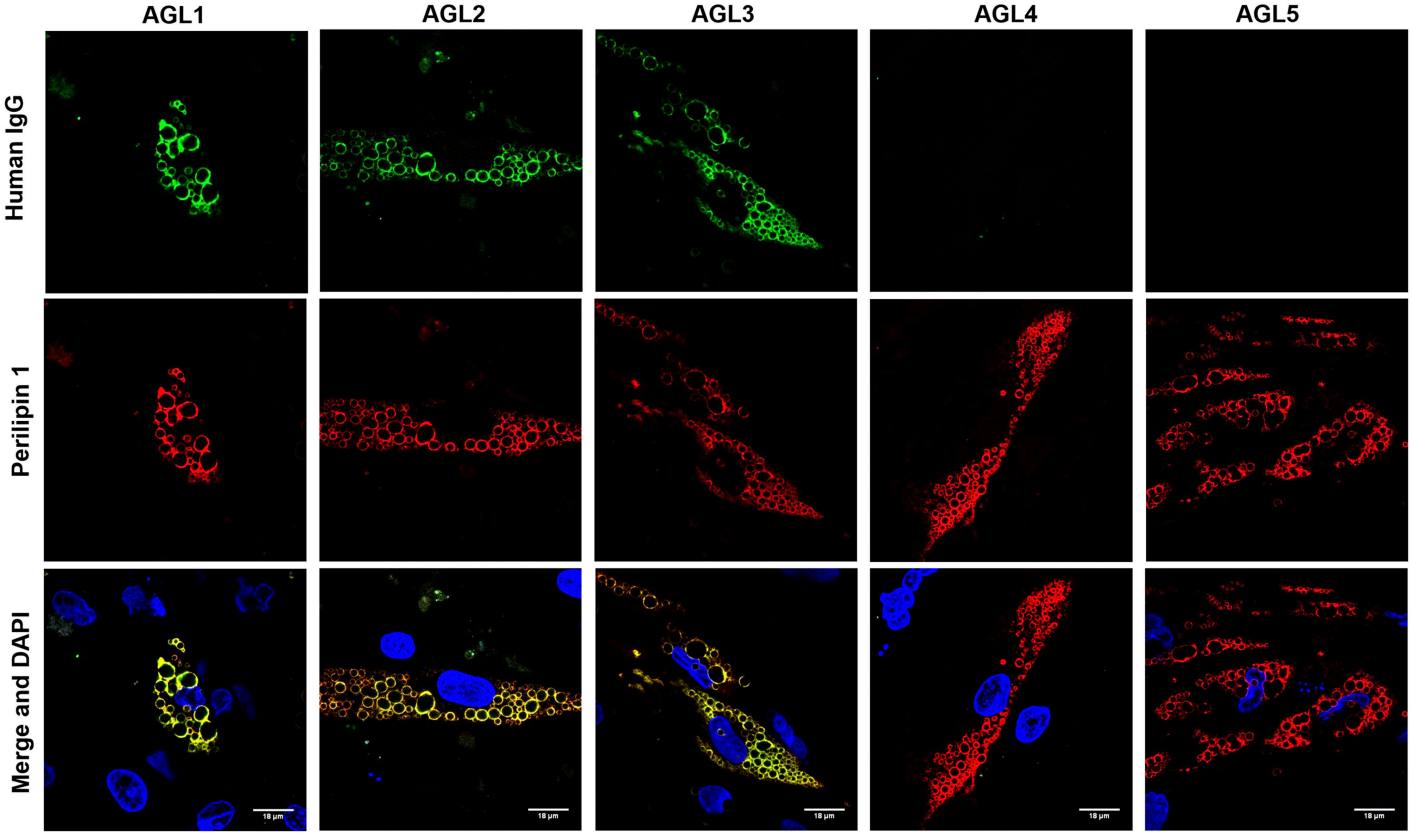
Colocalization of PLIN1 and anti-PLIN1 autoantibodies from patients with acquired generalized lipodystrophy. Confocal microscopic analysis of human preadipocytes revealed colocalization of PLIN1 and anti-PLIN1 IgG from AGL1 on the lipid droplet surface. However, no colocalization was observed when using serum from AGL4 and AGL5 (negative for anti-PLIN1 autoantibodies). DNA was stained with 4′,6-diamidino-2-phenylindole (DAPI, blue); IgG binding was detected using FITC-conjugated rabbit anti-human IgG (green); PLIN1 was detected with biotin-labeled rabbit IgG followed by Texas Red-labeled streptavidin (red). Scale bars correspond to 18 μm.

The perilipins (PLINs) comprise an ancient protein family defined by N-terminal sequence homology (17). Although PLIN1 was the foremost autoantigen candidate, other perilipins, such as PLIN2, PLIN3, and PLIN5, show high sequence homology with PLIN1. To demonstrate autoantibody specificity for PLIN1, we blocked AGL2 serum anti-PLIN1 IgG by preincubation with an excess of recombinant human PLIN1. Upon blockade, a significant reduction in the western blot signals (as compared with untreated samples) was observed (Figure 3D). In support of these data, confocal analyses demonstrated that IgG from patients AGL1, AGL2 and AGL3 colocalizes with PLIN1 on the lipid droplet. In contrast, no PLIN1/IgG colocalization was detected with IgG from healthy donors and patients with SLE, I-LA, or APL (Figure 4 and Figure S2).

**Figure 5:**
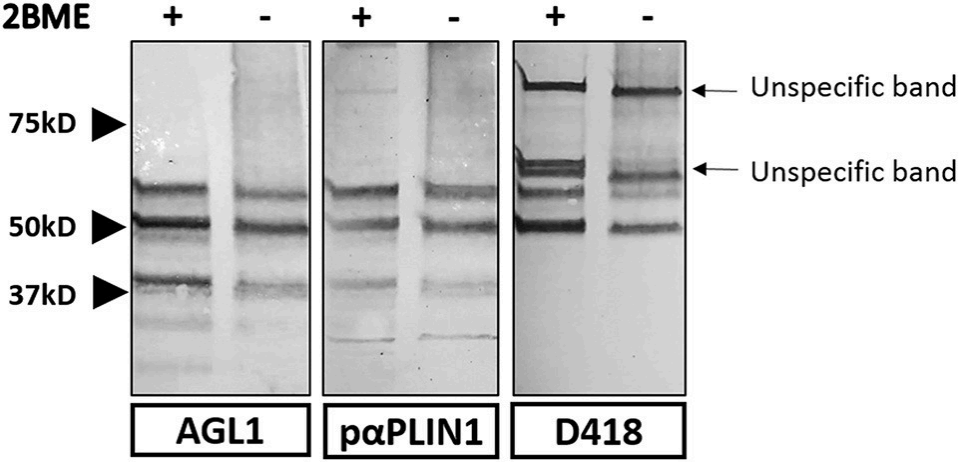
Strategy used for the characterization of the domains recognized by anti-PLIN1 autoantibodies. Results of a western blot screening performed on human adipose tissue extracts, under reducing (2BME +) or non-reducing (2BME –) conditions, incubated with serum sample from one patient with acquired generalized lipodystrophy (AGL1), or two polyclonal anti-PLIN1 antibodies directed against different regions of the protein. PαPLIN1 corresponds to antibody #AF6615 from R&D. D418 corresponds to the results obtained using a rabbit polyclonal anti-PLIN1 antibody targeting the C-terminal domain. Arrows indicate unspecific binding by the secondary antibody.

In order to study the target domain of PLIN1 recognized by the anti-PLIN1 autoantibodies, we performed a western blot assay using a different polyclonal anti-PLIN1 antibody specifically binding to the C-terminal domain (D418). This antibody detected the 60 and 55 kDa, but no the 37 kDa bands, in contrast with the reactivity obtained using the serum from AGL1 and the polyclonal anti-PLIN1 antibody (#AF6615) (Figure 5).

### Anti-PLIN1 autoantibodies increase basal lipolysis in cultured adipocytes

**Figure 6:**
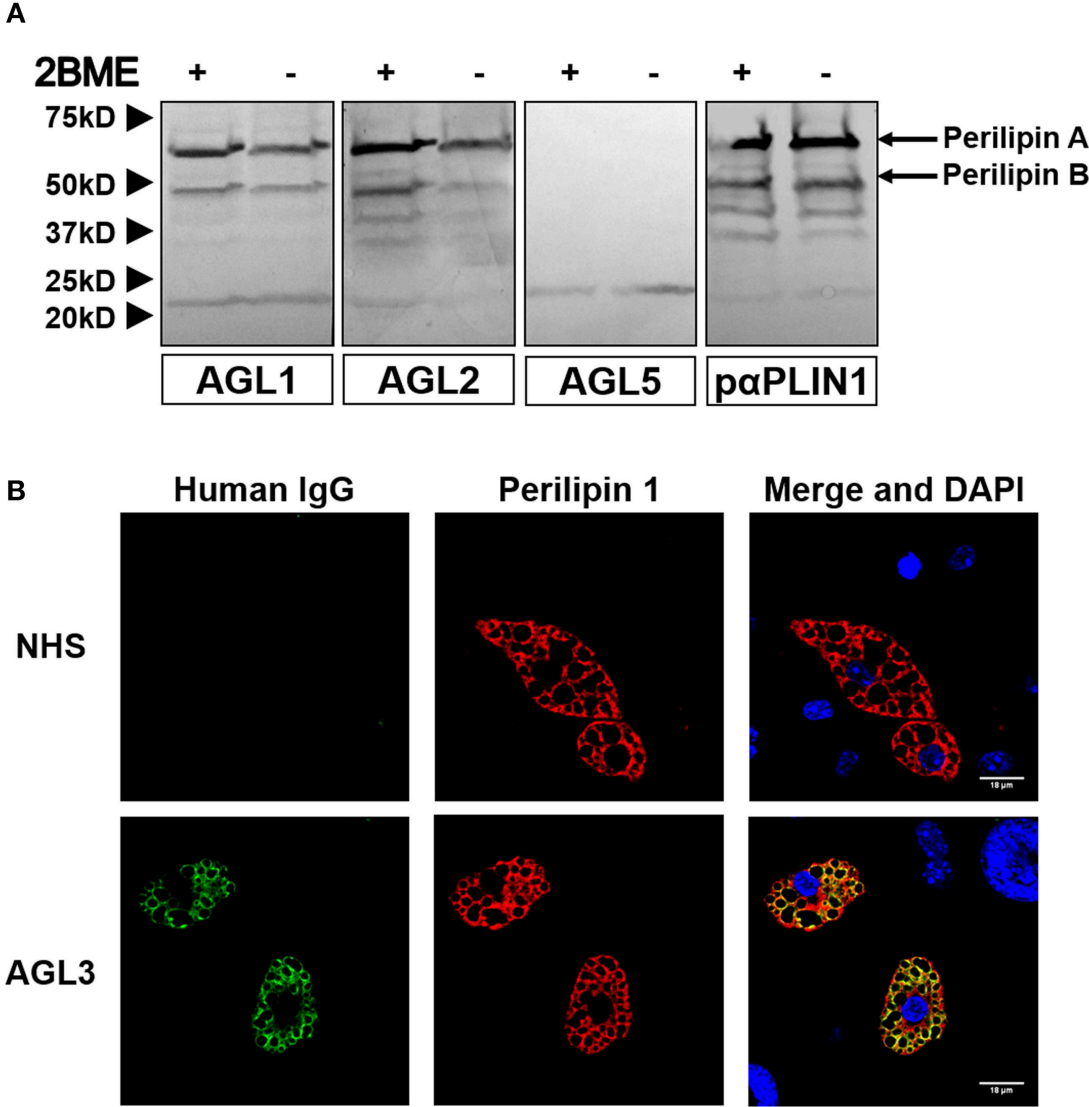
Mouse PLIN1 detection using serum from patients with AGL. **(A)** Tissue extracts were blotted with sera (1:100 dilutions) from patients with AGL known to be reactive (AGL1 and AGL2) or non-reactive (AGL5) with human PLIN1. Reactive sera from patients with AGL detect the two major isoforms of mouse PLIN1 (called PLINA and PLINB). As a positive control, the extract was blotted with a rabbit polyclonal anti-PLIN1 antibody. **(B)** Confocal microscopic analysis of mouse preadipocytes revealed colocalization of PLIN1 and IgG from patient AGL3 on the lipid droplet surface (merged image, in yellow). However, no colocalization was observed when using serum from one healthy donor (NHS). DNA was stained with 4′,6-diamidino-2-phenylindole (DAPI, blue); IgG binding was detected using FITC-conjugated rabbit anti-human IgG (green); PLIN1 was detected with biotin-labeled rabbit IgG followed by Texas Red-labeled streptavidin (red). Scale bars correspond to 18 μm.

**Figure 7:**
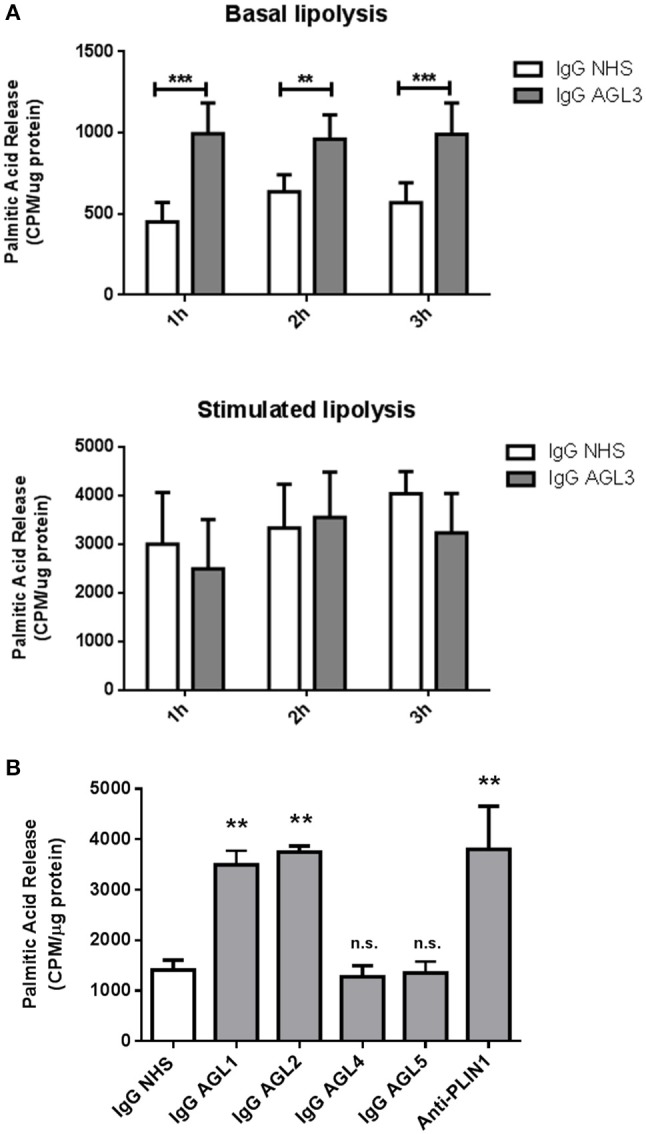
Functional effects of anti-PLIN1 autoantibodies on lipolysis. Preadipocytes (3T3-L1) were incubated overnight with [^3^H]palmitic acid at 37°C. **(A)** Results of the radiometric assessment of basal and stimulated lipolysis after 1, 2, and 3 h in preadipocytes treated with 300 micrograms of IgG purified from one patient with acquired generalized lipodystrophy (AGL3) with anti-PLIN1 autoantibodies and a healthy donor (NHS). Cells treated with IgG from patient AGL3 showed a significant increase in [^3^H]palmitic acid release under basal (^**^P < 0.01 at 2 h; ^***^P < 0.001 at 1 and 3 h) compared with cells treated with NHS IgG. Data represent mean ± SD, for triplicates of two independents experiments for each sample. Statistical significance was assessed with two-way ANOVA assay. **(B**) Radiolabeled palmitic acid release under basal conditions in preadipocytes treated with 300 micrograms of IgG purified from patient's AGL1, AGL2, AGL4 and AGL5 sera and a NHS. A commercial human PLIN1 Antigen Affinity-purified Polyclonal Antibody (10 μg per well) was used as positive control for blocking lipolysis regulation. Under these conditions, IgG purified from patients AGL1 and AGL2 induced a significant increase of basal lipolysis (^**^P < 0.01), comparable to that induced by a polyclonal anti-PLIN1 antibody (#AF6615 from R&D). Data represent mean ± SD, for triplicates of two independents experiments for each sample. Statistical significance was assessed with one-way ANOVA assay comparing the mean of each column with the mean of a control column (IgG NHS). n.s., not significant.

We hypothesized that the presence of anti-PLIN1 autoantibodies in patients with AGL could contribute to the phenotype of fat mass loss due to functional hindrance of perilipin. To explore this possibility, we assessed the effect of purified IgG from patients with AGL on the lipolytic activity of murine adipocytes. In this experimental setting, the 3T3-L1 cell line was chosen instead of hMSCs due to their higher differentiation rate. First, by means of indirect immunofluorescence, we verified that serum from patients with AGL recognized mouse PLIN1 in mice adipose tissue extracts (Figure 6A) and differentiated preadipocytes (Figure 6B). Subsequently, the basal and isoproterenol-stimulated lipolysis of cultured preadipocytes was measured by detecting the radiolabeled palmitic acid release to the medium. Cultured adipocytes were treated with 300 μg of purified IgG and lipolysis was measured at 60, 120, and 180 min thereafter. Cells treated with IgG from patient AGL3 showed a significant increase in [^3^H]palmitic acid release under basal, but not stimulated, conditions at all experimental times (P < 0.01 at 2 h; *P* < 0.001 at 1 and 3 h) compared with cells treated with healthy donor IgG (Figure 7A). In another experimental setting measuring end-point release of [^3^H]palmitic acid after 3 h in basal conditions, a commercial anti-PLIN1 polyclonal antibody (#AF6615) was used as positive control to block lipolysis regulation. In these conditions, cells treated with IgG from the remaining AGL patients (AGL1 and AGL2) exhibited a significant increase of basal lipolysis (*P* < 0.01), comparable to that induced by a polyclonal anti-PLIN1 antibody (Figure 7B).

### Clinical features associated with anti-PLIN1 autoantibodies

We examined the clinical characteristics of five patients with AGL, one male and four females (Table 1). In this cohort, three individuals presented the autoimmune variety of the disease while the remaining cases were classified as idiopathic. The lipodystrophy onset happened during childhood except for AGL5 in whom lipodystrophy appeared after withdrawal of oral contraceptives at the age of 31. Anti-PLIN1 autoantibodies were detected in the patients with the autoimmune variety of the disease (AGL1, AGL2, and AGL3). AGL1 developed type 1 diabetes mellitus during childhood, and at the moment of this study she had just started insulin treatment. Moreover, despite an increase of transaminases (ALT and AST) levels, she has not yet been diagnosed with fatty liver disease. AGL2 presented an elevation of transaminases and clinical signs of insulin resistance (hyperinsulinemia and the detection of anti-glutamic acid decarboxylase antibody), although he has not developed diabetes so far. AGL3 suffered severe metabolic disturbances like hyperinsulinemia and hepatic steatosis with a marked elevation of circulating triglycerides and transaminases (ALT and AST).

## Discussion

This study provides the first evidence for the presence of autoantibodies against the lipid droplet protein PLIN1 in patients with AGL. To date, the only description of antibodies against adipose tissue in human lipodystrophies was presented by Hübbler et al. (4). In that work, the authors established that IgG from one patient with AGL was able to react with adipocytes as judged by indirect immunofluorescence. Although the staining pattern provided by Hübler and colleagues was similar to that obtained by us (Figure 1B), they assumed, without further evidence, that antibody binding occurred on the plasmatic membrane instead of the lipid droplet membrane.

PLINs are an ancient family of proteins evolutionarily conserved from insects to mammals (18). While sharing sequence homology and the ability to bind lipid droplets, they differ in their sizes, tissue expression profiles, transcriptional regulation and affinities for lipid droplets. Depending on their expression patterns, PLINs can be classified into two groups: PLINs expressed in a tissue-restricted manner (PLIN1, PLIN4 and PLIN5) and those with ubiquitous expression (PLIN2 and PLIN3). Besides, from a mechanistic perspective, some PLINs are constitutively bound to lipid droplets (PLIN1 and PLIN2) while others exhibit exchangeable lipid droplet binding (PLIN3, PLIN4 and PLIN5) (18, 19). PLIN2, PLIN3, and PLIN5 differ from PLIN1 in their C-terminal sequences, which are considerably shorter (20).

PLIN1 is the founding and best characterized member of the perilipin family, with six phosphorylation sites (Ser-81, Ser-223, Ser-277, Ser-434, Ser-492, and Ser-517) (17, 21). Under basal (e.g., fed or insulin-stimulated) conditions, PLIN1 is unphosphorylated, and the two major lipolytic enzymes, adipose triacylglycerol lipase (ATGL) and hormone-sensitive lipase (HSL), are cytosolic and do not interact with lipid droplet-associated proteins. Moreover, via its C-terminal portion, unphosphorylated PLIN1 hijacks the ATGL activator comparative gene identification-58 (CGI-58), and lipolysis is suppressed (21). PLIN1 is available for rapid phosphorylation upon β-adrenergic stimulation. C-terminal phosphorylation of PLIN1 disrupts the interaction with CGI-58, allowing it to recruit, mobilize, and activate ATGL, which catalyzes the initial step of lipolysis. N-terminal phosphorylation of PLIN1 is essential for HSL recruitment on the lipid droplet surface (17, 19, 21). PLIN1 is expressed in adipocytes where it remains bound to the cytosolic lipid droplet and participates in the regulation of triacylglycerol storage and breakdown (17). Under basal conditions, PLIN1 restricts the access of cytosolic lipases to the neutral lipid core, thus promoting triacylglycerol storage, whereas upon β-adrenergic or adrenocorticotropic hormone stimulation, HSL is the major enzyme responsible of diglyceride hydrolysis (21). This is evidenced by the phenotype of PLIN1 null mice, which have a 65–70% reduction of adipose triacylglycerol storage compared with wild-type animals and are resistant to diet-induced obesity. Adipocytes isolated from perilipin null mice have a higher rate of basal lipolysis, which is consistent with the role for PLIN1 in protecting the core of the lipid droplet from the action of lipases. Strikingly, PLIN1 null mice also exhibit a reduced rate stimulated lipolysis due to the lack of a functional N-terminal PLIN1 domain responsible for HSL recruitment and diglyceride breakdown in the presence of the appropriate stimuli (22).

The consequences of defective lipid storage are dramatically apparent in rare human monogenic disorders of fat storage (lipodystrophies), in which the inability to store dietary lipids in the lipid droplets of specialized white adipose cells causes increased lipid deposits (ectopic fat) in nonadipose tissues, including skeletal and heart muscle, liver, and pancreas (17). FPLD4 is caused by autosomal dominant C-terminal mutations in PLIN1. When expressed in preadipocytes, human mutant PLIN1 proteins are associated with smaller lipid droplets, reduced triacylglycerol accumulation, and increased basal lipolysis. These *in vitro* results were concordant with morphometric analyses of adipose tissue in patients with FPLD4, showing smaller adipocytes than those of unaffected individuals (9, 10, 23). Interestingly, reduced expression of mutant PLIN1 in FLPD4 patients induces overexpression of PLIN2, which can in no way restore lipolysis regulation and normal phenotype due to the lack of a full C-terminal region in these PLIN homolog (10).

In our study, anti-PLIN1 autoantibodies detected in patients with AGL were found to increase basal lipolysis in cultured preadipocytes (Figure 7). Previous reports involving animal models have shown that fat loss can be caused by antibodies against adipose tissue (24–29). As demonstrated by Cheng et al. administration of a monoclonal anti-adipocyte antibody causes a reduction in body fat mass by suppressing subcutaneous adipose tissue development, reducing triglyceride biosynthesis, and promoting triglyceride lipolysis in adipose tissue (24). Although we did not investigate the *in vivo* mechanism of action of the anti-PLIN1 antibodies in AGL, it is conceivable that they might act by blocking key domains with a role in the attenuation of basal lipolysis. In order to map the epitope/s targeted by the anti-PLIN1 autoantibodies, we performed western blot assays with a different polyclonal anti-PLIN1 antibody specifically binding to the protein's C-terminal domain (D418) and found that it exclusively recognized the 60 and 55 kDa, but not the 37 kDa bands. Considering that the commercial polyclonal antibody #AF6615 and anti-PLIN1 autoantibodies equally recognize the 60, 55, and 37 kDa bands when blotted against healthy donors' adipose tissue extracts, this result is consistent with the 37 kDa band being a C-terminal truncated splicing variant of PLIN1 (Figure 5). The existence of a 37.5 kDa mouse splicing variant lacking C-terminal amino acids downstream to 422 (Uniprot Q8CGN5-3), also supports the 37 kDa band observed by us (Figure 1) representing the human splicing variant homologous to that short mouse isoform. However, in the absence of further studies, we cannot discard that such band represents a different autoantigen not related to PLIN1 and further research is being carried out to clarify this point. All considered, the most likely explanation is that the immune response inducing the appearance of anti-PLIN1 autoantibodies in patients with AGL was of polyclonal nature, thus targeting several epitopes throughout the protein. For example, binding of the autoantibody to the unphosphorylated C-terminal region could prevent the binding of CGI-58, thus allowing unrestrained lipolysis by ATGL during basal conditions, as demonstrated in perilipin null mice (22) and in patients with FPLD4 (9, 10, 23). However, the phenotype of fat mass loss in patients with AGL diverges from that characteristic of patients with FPLD4. These differences might be due to anti-PLIN1 autoantibodies blocking additional sites with relevant functions for PLIN1, for example the portion spanning amino acids 291–319, which is required for the interaction with CIDEC (30). Considering that stimulated lipolysis is determined by the N-terminal phosphorylation state and that the former is not affected by the autoantibody, the most likely interpretation of our results is that anti-PLIN1 autoantibodies bind to and block the central and C-terminal regions of the protein. To better support this hypothesis, further experiments are scheduled for the production of recombinant PLIN1 fragments in order to map the epitope/s recognized by anti-PLIN1 autoantibodies using immunochemical tools. Moreover, the availability of PLIN1 peptides will allow mice immunization and the generation of anti-PLIN1 antibodies specifically targeting amino- or carboxi-terminal domains. Finally, functional assessment of these autoantibodies on basal lipolysis could be eventually carried out using cultured preadipocytes.

In our cohort of five patients with AGL, three presenting the autoimmune variety of the disease (Table 1) had antibodies against PLIN1, whereas idiopathic cases were negative. The observed frequency of autoantibodies is most probably biased because of the low proportion of idiopathic cases and the absence of panniculitis-associated phenotypes in the cohort. Indeed, in our view, antibodies directed against adipose tissue should also be investigated in panniculitis-associated AGL cases, since this presentation of the disease is typically accompanied by autoimmune diseases (although at lower frequencies than autoimmune AGL).

We describe here the finding of autoantibodies against PLIN1, one of the most abundant proteins on the lipid droplet surface of mature white adipocytes, in the serum of three patients with the autoimmune variety of AGL. As a result, anti-PLIN1 IgG autoantibodies from patients were able to induce a significant elevation of basal lipolysis in cultured mouse 3T3-L1 preadipocytes. Our data provide strong support for the conclusion that anti-PLIN1 autoantibodies are implicated in the pathogenesis of the generalized lipodystrophy in these patients. Despite the identification of anti-PLIN1 antibodies, we do not discard that autoimmune AGL patients may target other, additional autoantigens. As shown in other autoimmune pathologies, multiple antigens can be involved in a single disease process, whether as markers or casual factors (31, 32). Further research, probably with a more throughput approach, is needed to identify other possible antigens and their involvement in AGL. The finding of anti-PLIN1 autoantibodies in patients with AGL sheds light on the pathophysiology of the disease, provides a target for its difficult diagnosis and opens the possibility for novel therapeutic strategies.

## Author contributions

FC, ML-T, and MdM conceived, designed and supervised the studies. FC, VA, SG, and AL-L developed the experimental works. AL-L and DA-V gathered the clinical data. FC wrote the first draft of the manuscript and revised it with considerable input from ML-T, MdM, AL-L, and DA-V.

### Conflict of interest statement

The authors declare that the research was conducted in the absence of any commercial or financial relationships that could be construed as a potential conflict of interest.

## Abbreviations

AGL: acquired generalized lipodystrophy
APL: acquired partial lipodystrophy
ATGL: adipose triacylglycerol lipase
CGI-58: comparative gene identification-58
FPLD4: familial partial lipodystrophy type 4
hMSCs: human mesenchymal stem cells
HSL: hormone-sensitive lipase
IBMX: isobutylmethylxanthine
I-LA: localized lipoatrophy due to intradermic insulin injections
PLIN: perilipin
SLE: systemic lupus erythematous.
