# Supplementary material for: Autoantibodies Against Perilipin 1 as a Cause of Acquired Generalized Lipodystrophy
Source: Front Immunol. 2018 Sep 19;9:2142. doi: 10.3389/fimmu.2018.02142 (PMC6156147; doi:10.3389/fimmu.2018.02142)
Supplement: Supplementary file 1 [file Data_Sheet_1.pdf]

SUPPLEMENTARY MATERIAL

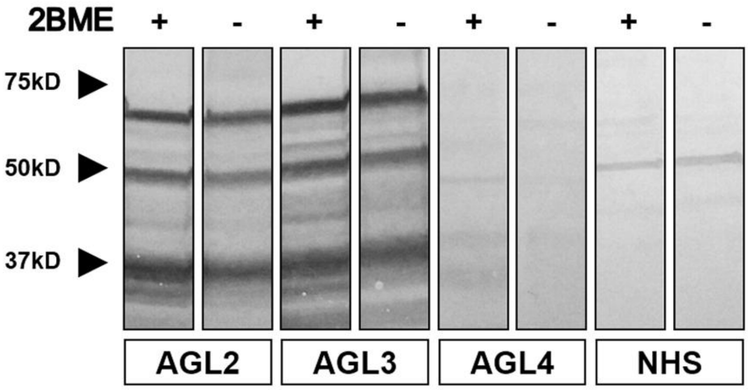

Figure S1. Reactivity to adipose tissue extract from another donor. Results of a western blot screening performed on human adipose tissue extracts, under reducing (2BME +) or non-reducing (2BME -) conditions, incubated with serum samples from three patients with acquired generalized lipodystrophy (AGL2, AGL3 and AGL4) and one healthy donor (NHS). AGL2 and AGL3 showed a similar reactivity that with the extract presented in figure 1 of the main text.

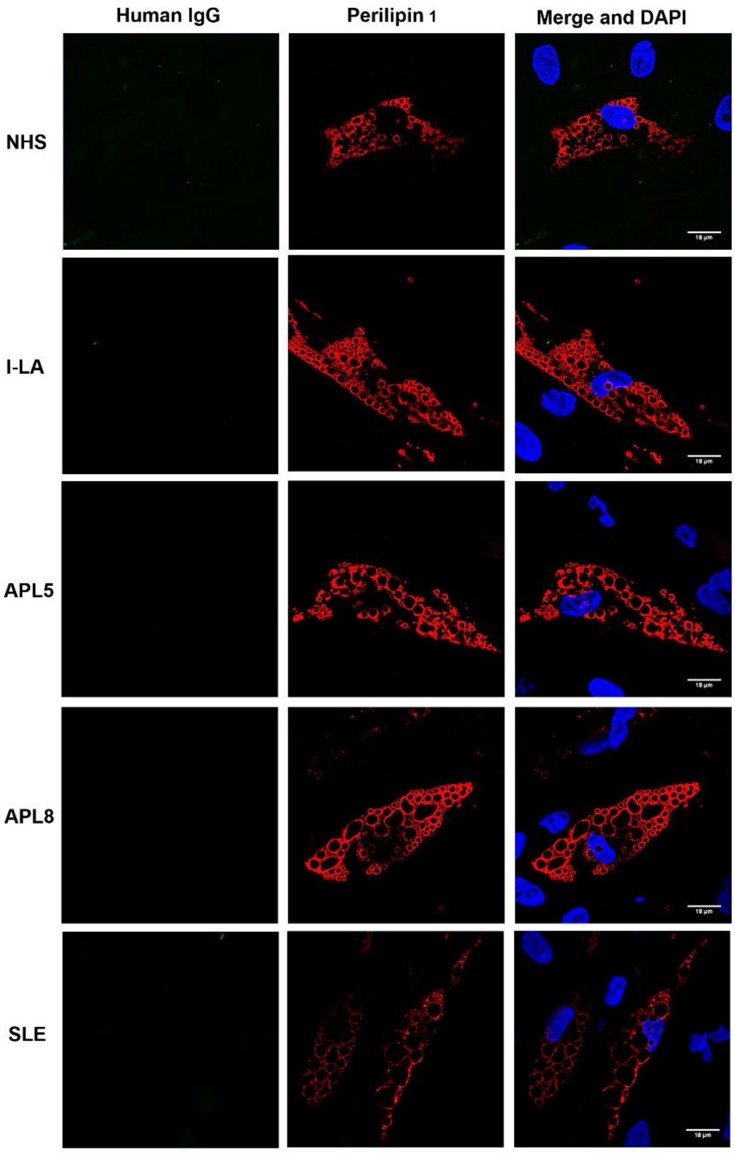

Figure S2. No colocalization of PLIN1 and human serum IgG from control samples. Confocal microscopic images are presented to supplement Fig. 4 of the main text. Fixed and permeabilized cultured human preadipocytes were incubated with serum samples (1:200 dilutions) and a rabbit monoclonal anti-human PLIN1 antibody (1:100 dilutions). Confocal microscopic analysis of human preadipocytes revealed no colocalization when using one healthy donor (NHS), one patient with localized lipoatrophy (I-LA), two patients with acquired partial lipodystrophy (APL) or one patient with Systemic Lupus Erythematosus (SLE). DNA was stained with 4’,6-

diamidino-2-phenylindole (DAPI, blue); PLIN1 was detected with biotin-labeled rabbit IgG followed by Texas Red-labeled streptavidin (red). Scale bars correspond to 18 μm.
